# *Enterobacterales* Infection after Intestinal Dominance in Hospitalized Patients

**DOI:** 10.1128/mSphere.00450-20

**Published:** 2020-07-22

**Authors:** Krishna Rao, Anna Seekatz, Christine Bassis, Yuang Sun, Emily Mantlo, Michael A. Bachman

**Affiliations:** a Department of Internal Medicine, Division of Infectious Diseases, University of Michigan, Ann Arbor, Michigan, USA; b Department of Pathology, University of Michigan, Ann Arbor, Michigan, USA; JMI Laboratories

**Keywords:** *Enterobacterales*, infection prevention, microbiome, prognostic indicators

## Abstract

The *Enterobacterales* order of Gram-negative bacteria includes the common nosocomial pathogens Klebsiella pneumoniae, Escherichia coli, Serratia marcescens, and *Enterobacter* species. Intestinal domination by some colonizing bacterial taxa is associated with subsequent infection, but 16S rRNA gene sequencing is too costly and slow to be used in a clinical setting. The objectives of this study were to develop a PCR-based assay that can measure *Enterobacterales* density, validate it against 16S rRNA gene sequencing, and measure the association between *Enterobacterales* dominance and subsequent infection. Two quantitative PCR (qPCR) assays that were developed to quantify the absolute and relative abundance of *Enterobacterales* had good correlation with 16S rRNA sequence analysis (*P <* 0.0001). Using both PCR assays and 16S sequencing, a matched case-control study was performed comparing rectal swabs from hospitalized patients who later developed bloodstream, urinary tract, or respiratory *Enterobacterales* infections (*n* = 95) to swabs from patients who remained uninfected (*n* = 189). *Enterobacterales* abundance measured by sequencing was high in both cases and controls (means, 31.1% and 27.5%, respectively; *P =* 0.322). We observed an increased risk of infection that depended on both the absolute and relative abundance of *Enterobacterales* as measured by qPCR assay A (*P =* 0.012). After adjustment for albumin levels, central venous catheter presence, and use of cephalosporins at the time of swab collection, this association still approached significance (*P =* 0.061). These results demonstrate that using qPCR to measure intestinal colonization dominance is feasible, indicate that hospitalized patients have high levels of *Enterobacterales* colonization, and suggest that both relative and absolute abundance may be associated with subsequent infection.

**IMPORTANCE** Increasing antibiotic resistance has resulted in infections that are life-threatening and difficult to treat. Interventions that prevent these infections, particularly without using antibiotics, could save lives. Intestinal colonization by pathogens, including vancomycin-resistant *Enterococcus* and carbapenem-resistant *Enterobacteriaceae* (part of the order *Enterobacterales*) is associated with subsequent infection, and increased colonization density is associated with increased infection risk. Therefore, colonization offers a window of opportunity for infection prevention if (i) there are rapid and inexpensive assays to detect colonization, (ii) there are safe and effective interventions, and (iii) the risk of infection outweighs the risk of the treatment. Fecal transplants are proof of principle that manipulating the microbiome can reduce such colonization and prevent infections. This study demonstrates the feasibility of implementing rapid and inexpensive assays to quantify colonization and measures the strength of association between *Enterobacterales* dominance and subsequent infection. The approach described here could be a valuable tool in the prevention of antibiotic-resistant infections.

## Abstract (precis)

Increasing antibiotic resistance has resulted in infections that are life-threatening and difficult to treat. Interventions that prevent these infections, particularly without using antibiotics, could save lives. Intestinal colonization by pathogens, including vancomycin-resistant *Enterococcus* and carbapenem-resistant *Enterobacteriaceae* (part of the order *Enterobacterales*) is associated with subsequent infection, and increased colonization density is associated with increased infection risk. Therefore, colonization offers a window of opportunity for infection prevention if (i) there are rapid and inexpensive assays to detect colonization, (ii) there are safe and effective interventions, and (iii) the risk of infection outweighs the risk of the treatment. Fecal transplants are proof of principle that manipulating the microbiome can reduce such colonization and prevent infections. This study demonstrates the feasibility of implementing rapid and inexpensive assays to quantify colonization and measures the strength of association between *Enterobacterales* dominance and subsequent infection. The approach described here could be a valuable tool in the prevention of antibiotic-resistant infections.

## INTRODUCTION

Colonization with *Enterobacterales* species, an order encompassing the *Enterobacteriaceae* family, including Escherichia coli, *Klebsiella*, and *Enterobacter*, and other families of Gram-negative pathogens (1), has been associated with subsequent infection, which is both a challenge and opportunity for infection prevention. As an example, for *Klebsiella*, 5.2% of colonized patients became infected compared to 1.3% of noncolonized patients (2). This association appears stronger in antibiotic-resistant *Enterobacteriaceae*—based on a review of 10 studies, carbapenem-resistant *Enterobacteriaceae* (CRE) infections occurred in 16.5% of colonized, hospitalized patients (3). Increasing colonization density may increase the risk of subsequent infection. In patients undergoing stem cell transplantation, a relative abundance of *Proteobacteria* above 30% was associated with subsequent Gram-negative bacteremia (hazard ratio, 5.2) (4). In long-term acute care patients, a relative abundance of CRE Klebsiella pneumoniae above 22% was associated with a relative risk of 4.2 for subsequent infection (5). This association of colonization with subsequent infection provides a window of opportunity for intervention with emerging therapies, including decolonization by antibiotics or manipulation of the microbiome. In fact, in 142 patients across 23 reports who underwent fecal transplant for decolonization of multidrug-resistant organisms (MDROs), 78% achieved microbiological clearance (6), and case reports suggest interruption of MDRO infections (7).

Measuring colonization density could be an effective tool for infection prevention if it could be deployed in clinical laboratories. To date, measures of colonization density have required massively parallel 16S rRNA gene sequencing that is expensive and slow. Quantitative PCR (qPCR) assays could be a rapid and less expensive approach to detect and quantify intestinal colonization by pathogens of concern in our hospitals. Since qPCR has become a standard tool in clinical microbiology, deploying these assays would not require additional expertise or equipment. To test the feasibility of qPCR measurement of colonization density and the association between *Enterobacterales* dominance and subsequent infection, we developed two qPCR assays, compared their performance to 16S sequencing, and assessed their association with subsequent *Enterobacterales* infection in a case-control study.

## RESULTS

### *In silico* analysis of primer and probe binding.

The TaqMan assays were designed to use an *Enterobacterales* probe and a broad-range bacterial probe targeting different regions of the same bacterial 16S rRNA gene amplicon (8, 9) (see Fig. S1A in the supplemental material). To do so, we identified a conserved region of the bacterial 16S rRNA gene amplicon in 35 clinically relevant *Enterobacterales* and designed two probes that accounted for a GG/AC polymorphism in this region. In combination, the two *Enterobacterales* probes exactly matched 274/319 *Enterobacterales* sequences using the Ribosomal Database Project’s Probe Match (85.9% [Table 1]). Outside the *Enterobacterales* order, the MGB_ENT_GG_Probe matched only 2,295/1,449,392 (0.2%) non-*Enterobacterales* and the MGB_ENT_AC_Probe matched only 22,455/1,449,392 (1.5%) non-*Enterobacterales*, even when expanding the search to also include nontype strains and uncultured bacteria. Most non-*Enterobacterales* matches were to *Gammaproteobacteria* (24,707/24,750 [99.8%]) but were not likely to be found in human gut samples (e.g., *Vibrionales*, *Oceanospirillales*). However, the MGB_ENT_AC_Probe did match many *Pasteurellales*, including *Haemophilus*, which can be found in human gut samples, although not normally at high levels. MGB_Uniprobe_p1, a shorter version of the UniProbe used by Yang et al. (8) had an exact match to 11,627/12,227 (95.1%) 16S sequences in Ribosomal Database Project (RDP) release 11.5 categorized as type strains, isolates, size ≥ 1,200, and good quality. The Forward primer A and Reverse primer A (8) did not match many *Bacteroidetes* sequences in the RDP database (62.2% and 36.3%, respectively, Table 1). Therefore, we designed a second assay using the same *Enterobacterales* probes but with primers matching a broader range of bacteria, including >90% *Bacteroidetes* (Forward primer B 1,194/1,315 [90.8%], Reverse primer B,1,256/1,315 [95.5%]) (Table 1 and Fig. S1B). The second assay also used a different universal probe, Uniprobe B, with an exact match to 10,975/12,736 (86.2%) bacterial sequences categorized as type strains, isolates, size ≥ 1,200, and good quality, including wide coverage of the major groups of human gut bacteria *Bacteroidetes*, *Firmicutes*, and *Proteobacteria*.

**TABLE 1:** Primers and probes used in this study

Oligonucleotide primer or probe^*a*^	Final Rx concn (nM)^*b*^	Sequence (5′ to 3′)	Assay	No. of exact sequence matches/total no. of sequences (%)^*c*^			
				*Enterobacterales*	*Proteobacteria* (non-*Enterobacterales*)	*Firmicutes*	*Bacterioidetes*
MGB_ENT_GG_Probe (FAM)	250	TACCTGGTCTTGACATCCA	Both	62/319 (19.4)	59/4,273 (1.4)	0/2,438 (0)	0/1,315 (0)
MGB_ENT_AC_Probe (FAM)	250	TTACCTACTCTTGACATCCA	Both	212/319 (66.4)	353/4,273 (8.3)	0/2,438 (0)	0/1,315 (0)
MGB_Uniprobe _p1 (VIC)	250	ACGAGCTGACGACARC	A	314/319 (98.4)	4,120/4,273 (96.4)	2,357/2,438 (96.7)	1,254/1,315 (95.4)
Uniprobe_B (VIC)	250	ATTGACGGGGRCCCGCACAAG	B	315/319 (98.7)	3,762/4,273 (88.0)	2,327/2,438 (95.4)	1,034/1,315 (78.6)
Forward primer A (P891F)	900	TGGAGCATGTGGTTTAATTCGA	A	312/319 (97.8)	3,214/4,273 (75.2)	16,83/2,438 (69.0)	818/1,315 (62.2)
Reverse primer A (p1022R)	900	TGCGGGACTTAACCCAACA	A	316/319 (99.1)	3,192/4,273 (75.3)	2,259/2,438 (92.7)	478/1,315 (36.3)
Forward primer B (F785)	900	GGATTAGATACCCTGGTAGTCC	B	316/319 (99.1)	4,138/4,273 (96.8)	2,214/2,438 (90.8)	1,194/1,315 (90.8)
Reverse primer B (R1097 – P2)	900	GAGCTGACGACARCCATGC	B	314/319 (98.4)	3,980/4,273 (93.1)	2,329/2,438 (95.5)	1,256/1,315 (95.5)

a FAM, 6-carboxyfluorescein.

b Rx, reaction.

c Matching 16S sequences from type strains, isolates, size ≥1,200, and good quality in RDP release 11.5.

10.1128/mSphere.00450-20.1 FIG S1 Locations of TaqMan assay primers (green arrows) and probes (red and blue bars) in relation to variable regions (gray) 5 and 6 of the bacterial 16S rRNA gene. (A) TaqMan assay A. AF, Forward primer A (P891F); AR, Reverse primer A (p1022R); ENT, locations of Ent AC MGB and Ent GG MGB probes. (B) TaqMan assay B. BF, Forward primer B (F785); BR, Reverse primer B (R1097 – P2). Download FIG S1, PDF file, 0.3 MB. Copyright © 2020 Rao et al. 2020 Rao et al. This content is distributed under the terms of the Creative Commons Attribution 4.0 International license.

### Assay optimization.

Despite differences in the 16S rRNA gene sequences between *Enterobacterales* and non-*Enterobacterales*, some cross-reactivity was observed in binding of the *Enterobacterales* probe. Although the amplification curves were not exponential, final fluorescence was strong enough to cross the fluorescence threshold used to quantify the PCR result. To increase specificity, a number of PCR additives were tried empirically. Addition of dimethyl sulfoxide (DMSO) (5% [vol/vol]) was able to decrease or eliminate reactivity with three non-*Enterobacterales*: Pseudomonas aeruginosa, Eubacterium rectale, and *Lachnospiraceae* (Fig. S2). This additive was used for all subsequent testing.

10.1128/mSphere.00450-20.2 FIG S2 DMSO (5%) increases specificity of *Enterobacterales* probe. For all amplification plots, *n* = 3. PMAxx was not used in this experiment. Download FIG S2, TIF file, 0.7 MB. Copyright © 2020 Rao et al. 2020 Rao et al. This content is distributed under the terms of the Creative Commons Attribution 4.0 International license.

PCR using universal primers can be confounded by contamination of the reaction mixture from the laboratory or the reagents themselves (10). Indeed, we observed reactivity in no-template control reactions. To eliminate this contaminating DNA, which might alter the quantification of total bacterial DNA, we added a proprietary derivative of propidium monoazide, PMAxx (Biotium, Fremont, CA; 6 μM) to the reaction mixture. PMA is an intercalating agent that binds readily to double-stranded DNA (dsDNA). Photolysis (460-nm-wavelength exposure) produces a nitrene that can then covalently bind to DNA, forming cross-links that prevent use as a PCR template (10–12). Addition of PMAxx eliminated reactivity seen in the no-template control while allowing robust amplification of a K. pneumoniae 16S template (Fig. S3).

10.1128/mSphere.00450-20.3 FIG S3 Addition of PMA eliminates reactivity in no-template controls. For No Template (water), *n* = 2; for K. pneumoniae 16S plasmid, *n* = 3. DMSO (5%) was used for each reaction. Download FIG S3, TIF file, 0.5 MB. Copyright © 2020 Rao et al. 2020 Rao et al. This content is distributed under the terms of the Creative Commons Attribution 4.0 International license.

### Linearity, precision, and reportable range.

To compare the linearity and reportable ranges of assay A and assay B, serial dilutions of various templates were performed, and linear regression was performed based on input DNA and cycle threshold (*C_T_*) values (Table 2). Both assays were linear, although the slopes were closer to −3.3 for assay A, which would represent perfect PCR efficiency, and closer to each other for the Ent probe and the universal probe. Since the Ent probe is a mixture containing a GG dinucleotide (binds to K. pneumoniae among others) and AC (binds to Serratia marcescens among others), we tested linearity and reportable range in serial dilutions of S. marcescens genomic DNA (Table 2). Again, the slope for assay A was closer to −3.3 than assay B.

**TABLE 2:** Linearity of qPCR assays

Assay	Serial dilution sample	*Enterobacterales* probe (Ent)			Universal probe (Uni)		
		Range (log_10_ CFU)	Slope	*r* ^2^	Range (log_10_ CFU)	Slope	*r* ^2^
A	K. pneumoniae	3–9	−3.441	>0.99	3–9	−3.374	>0.99
	S. marcescens	3–8	−3.46	>0.99	3–8	−3.46	>0.99
	K. pneumoniae, E. coli, S. marcescens, Citrobacter freundii, and Enterobacter cloacae	3–9	−3.371	>0.99	3–9	−3.282	>0.99

B	K. pneumoniae	3–9	−3.647	>0.99	3–9	−3.554	>0.99
	S. marcescens	4–8	−3.594	>0.99	4–8	−3.683	>0.99
	K. pneumoniae, E. coli, S. marcescens, Citrobacter freundii, and Enterobacter cloacae	3–9	−3.529	>0.99	3–9	−3.33	>0.99

**FIG 1:**
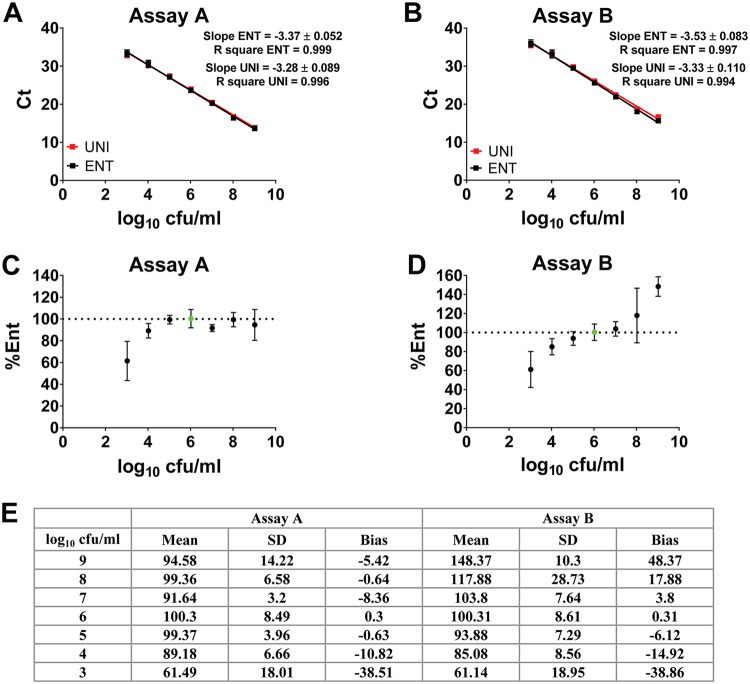
Linearity and reportable range of assays A and B on a mixture of five *Enterobacterales* species (K. pneumoniae, E. coli, S. marcescens, Citrobacter freundii, and Enterobacter cloacae). (A and B) Extracted genomic DNA mixtures in a serial dilution were amplified by assay A (A) and assay B (B) with six technical replicates. (C and D) Percentages of *Enterobacterales* based on assay A (C) and assay B (D) based on the dd*C_T_* method relative to the log_10_ 6 CFU/ml value (green). (E) Mean, standard deviation (SD), and bias of percent *Enterobacterales* calculated from assay A (C) and assay B (D).

Rectal swabs are likely to contain mixtures of *Enterobacterales* with both the AC and GG polymorphisms in the probe target sequence. To assess linearity and reportable range from a mixture of *Enterobacterales*, overnight cultures of K. pneumoniae, E. coli, S. marcescens, Citrobacter freundii, and Enterobacter cloacae were mixed in equal volumes, serial dilutions were made, and six replicates were tested by assays A and B. As seen for individual bacteria, assay A had a slope close to −3.3 for both Ent and Uniprobe, whereas for assay B, the Ent probe slope (−3.5) diverged from the Uniprobe slope (−3.3; Fig. 1A and B; Table 2).

To calculate percent *Enterobacterales*, the delta-delta-*C_T_* (dd*C_T_*) method was used, calculating the ratio of Ent and Uniprobe *C_T_* values and using the log_10_ 6 CFU/ml samples as the value representing 100% *Enterobacterales*. Assay A did not show significant bias with changing concentrations and showed good precision (standard deviation [SD] < 10) between log_10_ 4 to 8 CFU/ml (Fig. 1C and E). In assay B, there was proportional bias in the calculated percent *Enterobacterales* with overestimation at higher total CFU and underestimation at lower CFU, likely attributable to the differences in PCR efficiency slopes (Fig. 1D; Table 2). The precision was <10 between log_10_ 4 to 7, corresponding with the lowest bias (Fig. 1D and E).

The bacterial population on the rectal swabs will be a mixture of *Enterobacterales* and non-*Enterobacterales* species, and the total fecal content may vary from swab to swab. To determine the linearity and reportable range based on total bacterial CFU, the five *Enterobacterales* species were mixed with two non-*Enterobacterales*, Bacteroides ovatus and Eubacterium rectale. For the qPCR experiments, the percent *Enterobacterales* was calculated with the mean d*C_T_* from positive-control K. pneumoniae and S. marcescens genomic DNA (gDNA) defined as 100%. All assays were internally consistent in percent *Enterobacterales* calculated down to a total bacterial CFU of log_10_ 5 CFU/ml (Fig. S4 [the dashed line represents the lower limit of reportable range]). Taken together, the linearity and precision data above indicate that the reportable range of qPCR assays A and B are a total bacterial concentration of log_10_ 5 to 9 CFU/ml of sample. This corresponded to an average Uniprobe *C_T_* of 14.5 to 30.1 for assay A and 16.6 to 32.6 for assay B.

10.1128/mSphere.00450-20.4 FIG S4 Linearity and reportable range of % *Enterobacterales* calculated by 16S and qPCR from serial dilutions of mixtures of five *Enterobacterales*, *B. ovatus*, and E. rectale. The mixture was made and diluted serially, split into three replicates, and DNA extraction, 16S sequencing, and PCR using assay A and B was performed. For the qPCR experiments, the % *Enterobacteriaceae* was calculated with the mean d*C_T_* from positive-control K. pneumoniae and S. marcescens gDNA defined as 100%. Download FIG S4, TIF file, 0.2 MB. Copyright © 2020 Rao et al. 2020 Rao et al. This content is distributed under the terms of the Creative Commons Attribution 4.0 International license.

### Accuracy.

**FIG 2:**
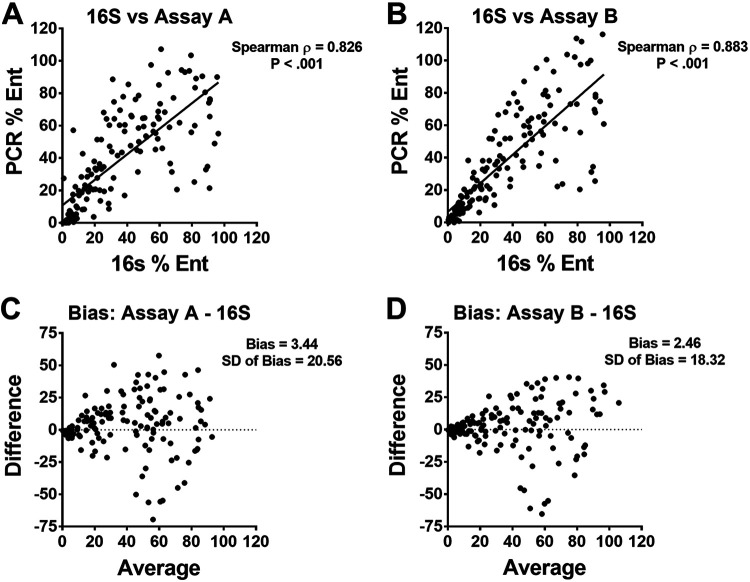
Accuracy of qPCR assays compared to 16S rRNA gene sequencing from 150 rectal swabs. (A) Percentages of *Enterobacterales* measured by 16S sequencing compared to assay A (A) and assay B (B). Bland-Altman plot comparing 16S sequencing to assay A (C) and assay B (D).

To assess the accuracy of each PCR assay compared to 16S rRNA gene sequencing (V4 region), we extracted DNA and performed sequencing and PCR assays A and B on patient rectal swabs. To test for detection of samples with known *Enterobacterales*, we included 50 rectal swabs that were culture positive for K. pneumoniae. Additionally, we included 100 samples that were culture negative for K. pneumoniae. The percent *Enterobacterales* of the 150 samples by 16S sequencing ranged from 0.332% to 96.2%. For qPCR analysis, K. pneumoniae genomic DNA was used as the calibrator to define the d*C_T_* that corresponds to 100% *Enterobacterales*. Spearman correlation was ρ = 0.826 (*P* < 0.01) for assay A and ρ = 0.883 (*P* < 0.01) for assay B (Fig. 2A and B). The bias was +3.4 for assay A and +2.5 for assay B (Fig. 2C and D). Despite the predicted differences in inclusivity of the primers in each assay, the correlation between qPCR assays is excellent (*r* = 0.9486, 95% CI of 0.9291 to 0.9629, and *P* < 0.0001). Overall, these data indicate that both qPCR assays are highly correlated with 16S sequencing results in calculating percent *Enterobacterales* from rectal swab samples.

### Initial and unadjusted analyses using 16S data.

The data set from 284 patient samples consisted of 95 cases and 189 matched controls (there was one case that could be matched to only a single control; see Data Set S1 in the supplemental material). Infections were caused by E. coli (33), *Klebsiella* (25), *Enterobacter* (13), *Serratia* (9), *Proteus* (6) and *Citrobacter* (2). These infections included 21 bloodstream, 29 urinary tract, and 45 respiratory infections. We had 155 males (54.6%), and mean age was 57.9 years (SD ±16.3). Based on 16S rRNA gene sequencing (232 samples), the mean/median relative abundance of *Enterobacterales* in cases versus controls were 31.1%/22.9% and 27.5%/19.6%, with considerable overlap between the two groups. The unadjusted analysis did not show that the difference in means here was statistically significant (*P =* 0.322), and this was true even for other variable constructions such as dichotomization, which argued against a threshold effect in the overall cohort. Only eight patients in our cohort met criteria for neutropenia (absolute neutrophil count of <500 cells/μl), and this was not associated with infection risk (*P >* 0.99).

10.1128/mSphere.00450-20.6 DATA SET S1 Metadata for subjects and their samples used in this study. Data include sample identifier (ID), patient demographics, bacterial culture results for cases of infection, clinical variables used in modeling, strata assigned to cases and controls in 1:2 matching scheme, 16S and PCR results for *Enterobacterales* abundance, including imputed values, *C_T_* values from PCR, sequencing success or failure, and Sequence Read Archive (SRA) BioProject and accession numbers. Download Data Set S1, XLSX file, 0.05 MB. Copyright © 2020 Rao et al. 2020 Rao et al. This content is distributed under the terms of the Creative Commons Attribution 4.0 International license.

Turning to the subgroup analyses for the respiratory and urinary infections revealed a similar lack of statistically detectable association (*P =* 0.896 and 0.888, respectively). However, bloodstream infections did show an association when dichotomized at the 3rd quartile of 60%—having a percent *Enterobacterales* by 16S rRNA gene sequence analysis [%Ent] of >60% confers a 10.1-fold increased odds of bloodstream infection (odds ratio [OR], 10.1 [1.17, 85.59], *P =* 0.036).

### Initial and unadjusted analyses using PCR assays A and B.

To independently assess the performance of both PCR assays on the novel task of predicting *Enterobacterales* infection from rectal swabs, we evaluated both relative abundance calculations and cycle thresholds (*C_T_*) for the numerator (Ent) and denominator (uniprobe) components. For assays A and B, we observed a similar lack of significant differentiation between cases and controls by relative abundance (median/mean for cases versus controls 32.2%/35% versus 30.7%/33.8% for assay A [*P* = 0.8, *n* = 235] and 24.5%/35% versus 24.9%/33.5% for assay B [*P* = 0.794, *n* = 222]). However, for assay A, there was an interaction between the numerator *C_T_* and percent *Enterobacterales*, and when accounting for this, the association with infection became statistically significant (*P =* 0.012 for interaction as a whole). That is, the absolute and relative abundance of *Enterobacterales* interacted with each other additively to increase the risk of infection. As *C_T_* values are the number of PCR cycles required to amplify the target to a detectable level, a lower *C_T_* indicates a higher abundance of target in the sample. For a specific example, at an *Enterobacterales* relative abundance of 35%, for every five-cycle decrease in *C_T_* (i.e., 32-fold increase in absolute *Enterobacterales* abundance), there is an increased odds of infection (OR, 3.36 [1.88, 5.98]; *P =* 0.012). The denominator *C_T_* for assay A associated with infection—for every 5-unit decrease in *C_T_* (i.e., 32-fold increased total bacterial abundance), we see a 2.8-fold increased odds of invasive infection (OR, 2.8 [1.53, 5.2]; *P* < 0.001).

For assay B, percent *Enterobacterales* did not associate with infection (*P =* 0.794). However, the numerator *C_T_* did show a significant association, but not as strong as for assay A (OR, 1.35 [1.09, 1.67]; *P =* 0.007). The denominator *C_T_* value did associate with infection, but it was also not as strong as for assay A (OR, 1.9 [1.1–3.2]; *P =* 0.012). Thus, only PCR assay A was considered for multivariable modeling, presented below.

### Adjusted analyses using 16S rRNA sequence data.

Stepwise regression constructed an adjusted multivariable model that included %Ent calculated from 16S sequence data (Table 3). This failed to show an association between %Ent and infection (*P =* 0.229) but identified clinical variables, notably cephalosporin and pressor use, as putative predictors of infection. We also attempted to construct an adjusted model for the bloodstream infection subgroup; however, this was much more limited as a result of having only 21 cases. The only clinical variable retained in this model was the presence of a central line at baseline. After this adjustment, for those with >60% *Enterobacterales* dominance in the gut, there is a 7.28-fold increase in the odds of bloodstream infection of borderline significance (OR, 7.28 [0.81, 65.36]; *P =* 0.076).

**TABLE 3:** Multivariable model of infection using 16S data

Variable	OR	95% confidence interval	*P* value
Central line at baseline	2.27	1.20, 4.30	0.012
Baseline albumin (g/dl)	0.50	0.29, 0.85	0.010
Pressors at baseline	5.47	1.29, 23.22	0.021
Cephalosporins at baseline	3.59	1.26, 10.25	0.017
%Ent	1.01	1.00, 1.02	0.229

### Adjusted analyses using PCR assay A.

For adjusted analysis of PCR assay A, the interaction with numerator *C_T_* was carried forward. Denominator *C_T_* values were considered but did not make it into the final model and when forced in (either alone or with various interactions), were not significant and did not improve model fit (data not shown); thus, the denominator *C_T_* was not included in adjusted analyses. However, percent *Enterobacterales*, being a relative abundance metric, does implicitly carry some denominator information into the final model. Save for use of pressors at baseline, the same clinical variables as in the 16S analysis made it into the final model (Table 4). We observed an increased risk of infection that depended on both the absolute and relative abundance of *Enterobacterales* as assessed by PCR assay A.

**TABLE 4:** Multivariable model of infection using qPCR assay A

Variable	β coefficient^*a*^	Standard error	OR [CI]^*b*^	*P* value
Central line at baseline	0.909	0.396	2.48 [1.14, 5.4]	0.022
Baseline albumin (g/dl)	−0.829	0.324	0.43 [0.23, 0.82]	0.011
Cephalosporins at baseline	1.89	1.26	6.64 [1.85, 23.8]	0.017

%Ent (per 10% increase)	0.377	0.262		
*C_T_* (per 5-cycle decrease)	0.38	0.172		0.061
%Ent × *C_T_* interaction	0.133	0.074		

a Taken from the logit model. These can be interpreted as increased risk (positive coefficient) or decreased risk (negative coefficient).

b Values provided only for independent covariates (i.e., no interaction present). CI, confidence interval.

Given the interaction term, the percent *Enterobacterales* and *C_T_* cannot be interpreted independently, so for these variables β coefficients and standard errors are presented in the table in lieu of odds ratios and confidence intervals, and the *P* value is presented for both terms and the interaction together. The β coefficients can be interpreted as increased risk (positive coefficient) or decreased risk (negative coefficient), but for the interaction terms, the coefficients must be summed and interpreted at a specific level. As an example of how to interpret the interaction results, after adjustment for clinical confounders and at the average numerator *C_T_* of 25, every 10% increase in percent *Enterobacterales* increases the odds of infection 2.8-fold (OR, 2.83 [1.47, 5.44]; *P =* 0.061). Conversely, at a relative abundance of 35% *Enterobacterales*, every five-cycle decrease in *C_T_* (i.e., 32-fold increase in absolute *Enterobacterales* abundance), there is an increased odds of infection (Table 4; OR, 2.32 [1.21, 4.48], *P =* 0.061).

## DISCUSSION

The aims of this study were to develop a qPCR assay to measure *Enterobacterales* abundance on rectal swabs, validate this PCR assay against 16S rRNA sequence analysis as the gold standard, and measure the association between *Enterobacterales* abundance and subsequent infection. Towards these aims, we developed and validated two real-time PCR assays that measure *Enterobacterales* as a proportion of total bacterial 16S rRNA-encoding DNA in a sample. These assays are somewhat complementary, as assay A had better analytical performance in terms of precision, linearity, and reportable range, but assay B was inclusive of *Bacteroides* species. Compared to the percent *Enterobacterales* calculated from 16S sequencing as the gold standard, both assays correlated well with minimal bias in patient rectal swab samples. This was true both in specimens that were culture positive for *Enterobacterales* (K. pneumoniae) and in the specimens used in the case-control cohort. Although there were challenges in terms of eliminating contaminating DNA from reagents, minimizing probe cross-reactivity, and optimizing inclusivity, these results indicate that relative abundance of bacterial taxa in microbiome samples can be measured accurately by qPCR.

Measured by 16S sequencing, the relative abundance of *Enterobacterales* in rectal swabs had a limited association with subsequent *Enterobacterales* infection in our cohort of intensive care and hematology/oncology patients. Overall, there was no significant difference in relative abundance between cases and controls as measured by 16S sequencing or qPCR. In adjusted analysis, relative abundance calculated from 16S sequencing was not significantly associated with infection. Many patients had percent *Enterobacterales* values that met prior definitions of dominance (>30%) (4), suggesting that both cases and controls may have an abnormal microbiome. However, *Enterobacterales* of >60% was associated with bloodstream infection. With only 21 cases in our data set, this is a preliminary finding as the ability to adjust for clinical confounders was limited. Notably, though, this is consistent with prior findings that intestinal domination by *Proteobacteria* and certain *Enterobacterales* species is associated with subsequent Gram-negative bacteremia in allogeneic hematopoietic cell transplant patients (4, 13). The potential association with bloodstream infections outside of transplant patients would be important to explore further, as bacteremia and sepsis are devastating clinical outcomes.

Although these qPCR assays were designed to measure relative abundance, our findings indicate that absolute abundance is important information that can be obtained from qPCR but not from 16S sequencing and that both absolute and relative abundance are best considered together in assessing the risk of infection. In unadjusted analysis, there was an interaction between *C_T_* values, a measure of absolute abundance, and percent *Enterobacterales*. Accounting for this interaction revealed a significant association between assay A qPCR results and subsequent infection. The *C_T_* value of the denominator in assay A was also associated with infection in unadjusted analysis. The design of these assays may be critical, as assay B, which sacrificed some analytical performance for increased inclusivity, was less informative. It is also unclear to what degree this superior performance was contingent upon the infections studied and whether this would change for other infections, which should be explored in future research. Adjustment for clinical variables still supported an association between increasing gut *Enterobacterales* and subsequent *Enterobacterales* infection. Our final clinical model for adjustment of qPCR variables included the presence of a central line, cephalosporin antibiotics, and albumin as associated with subsequent infection. In this context, the denominator *C_T_* value did not improve the performance of the model, but the interacting variables of relative and absolute *Enterobacterales* abundance remained informative.

Compared to 16S sequencing that provides relative abundance, qPCR can provide at least two additional measurements of the microbiota in patient samples: the absolute abundance of particular taxa and the total microbiota density in the sample. This approach may have broader applications, as reduced gut microbiota density has been associated with inflammatory bowel disease and recurrent Clostridioides difficile infection and is reversed by fecal transplantation (14). If the overall gut microbiota density varies between patients, measurement of both relative and absolute abundance of specific taxa may be required to assess associations with human health and disease. qPCR assays that predict infections based on measurements of the microbiota could be translated into practice, since many clinical laboratories are proficient in the validation and implementation of these assays. In summary, these results demonstrate that using qPCR to measure intestinal dominance by specific bacterial taxa is feasible, provides additional information compared to 16S rRNA gene sequence analysis, and suggests that increased intestinal *Enterobacterales* levels are associated with subsequent infection.

## MATERIALS AND METHODS

### Case definitions.

We collected discarded swabs sent for vancomycin-resistant enterococcus (VRE) screening during a 3-month period from patients in the intensive care units (ICUs) and select wards (hematology, oncology, and hematopoietic stem cell transplant) at Michigan Medicine. We then observed whether patients developed a bloodstream, respiratory, or urinary infection from a member of the *Enterobacterales* within 90 days after swab collection (including infections that occurred after hospital discharge) and identified them as putative cases. The putative cases were confirmed via manual chart review by the study team to decide whether they met clinical criteria derived from the Infectious Diseases Society of America, National Health Safety Network, and/or the American Thoracic Society guidelines (15–19). Following this, cases were matched 1:2 to controls based on age (±5 years), gender, and number of days (±30) between swab collections (to account for any temporal trends). This study was approved by the University of Michigan Institutional Review Board.

### Samples for PCR and 16S rRNA gene sequence analysis.

Rectal swab cultures were collected using the ESwab liquid Amies collection and transport system (Copan Diagnostics, Inc., Murrieta, CA), which elutes the swab sample into 1 ml of liquid Amies medium. The Amies medium samples were retrieved from −80°C storage, thawed, and extracted for DNA as described in Text S1 in the supplemental material. 16S rRNA gene sequencing and PCR assays A and B (described below) were performed for each sample from the same extracted DNA. Klebsiella pneumoniae and Serratia marcescens were cultured overnight in Luria-Bertani broth. The following species and strain were selected for a simple contrived community representative of common gut inhabitants: Eubacterium rectale, Bacteroides ovatus, Roseburia intestinalis, and *Lachnospiraceae* strain DW28. Strains were grown anaerobically in a Coy vinyl anaerobic chamber on modified or enriched brain heart infusion (BHI) liquid medium until reaching an optical density of 0.5 to 1, at which point individually grown strains were combined into an aliquot consisting of 40% *Lachnospiraceae* strain DW28, 30% E. rectale, 15% R. intestinalis, and 15% B. ovatus (Text S1). This contrived community was combined with different ratios of K. pneumoniae for “spiked” community analysis. Individual, combined, and spiked communities were used for DNA extractions.

10.1128/mSphere.00450-20.5 TEXT S1 Supplemental methods. Additional details on the methods of 16S rRNA gene-based sequencing and medium preparation for growth of non-*Enterobacterales* strains. Download Text S1, DOCX file, 0.02 MB. Copyright © 2020 Rao et al. 2020 Rao et al. This content is distributed under the terms of the Creative Commons Attribution 4.0 International license.

### DNA extraction.

Samples were added to wells of the bead plate included in the MagAttract PowerMicrobiome DNA/RNA kit (catalog no. 27500-4-EP; Qiagen) (formerly known as the MoBio PowerMag microbiome DNA/RNA kit) designed to be used in conjunction with the automated liquid handling Eppendorf EPmotion 5075 TMX (catalog no. 960020033; Eppendorf). Bacterial cultures were extracted from 200 μl of culture. Rectal swab samples were extracted from 250 μl of liquid Amies transport medium in the E-swab transport system. Subsequent steps to DNA extraction were conducted following the manufacturer’s directions using the Eppendorf EPmotion 5075 TMX. The resulting DNA was aliquoted into two replicate plates of 50 μl each, one stored for archive and the other used to generate the 16S rRNA gene-based sequencing.

### Primer design and evaluation.

Candidate primer and probe assays were optimized using Visual OMP software (DNA Software, Ann Arbor, MI), which uses secondary structures and thermodynamics of DNA hybridization to predict the melting temperatures of primers and probes compared to those of unintended dimerization products and calculate the expected efficiency of the PCR. Primer and probe coverage and specificity were evaluated using the Ribosomal Database Project’s Probe Match (RDP database release 11, update 5) (2).

### PCR assay protocol.

To balance amplification efficiency and inclusivity of the universal probe, two assays (A and B) were designed. Real-time PCR was performed using primers (IDT) and probes (Thermo Fisher Scientific) with sequences and concentrations listed in Table 1 in combination with TaqMan Gene Expression Master Mix (Thermo Fisher Scientific), 5% dimethyl sulfoxide (DMSO) (Sigma), 6 μM PMAxx (Biotium) (a proprietary derivative of propidium monoazide), and 10 μl template in a final reaction volume of 50 μl. Prior to template addition, the reaction mixture was incubated for 10 min at room temperature and then treated in a Biotium PMA-Lite LED (light-emitting diode) photolysis device for 10 min. PCR conditions were 50°C for 2 min, 95°C for 10 min, and then 40 cycles with 1 cycle consisting of 95°C for 15 s and 62°C for 1 min on a QuantStudio 3 real-time thermocycler (Thermo Fisher Scientific). As positive controls, genomic DNA from Klebsiella pneumoniae was used for the MGB_ENT_GG_Probe, Serratia marcescens was used for the MGB_ENT_AC_Probe and Eubacterium rectale was used for MGB_Uniprobe_p1 and Uniprobe_B. DNA-free water (Qiagen) was used as a negative control.

### 16S rRNA gene sequencing and analysis.

For details of the 16S rRNA gene sequencing and analysis, see Text S1 in the supplemental material.

### Statistical methods.

**(i) Initial analyses.** Initial descriptive statistics such as measures of central tendency/spread and counts were used to examine closely the primary variables of interest (percent *Enterobacterales* by 16S rRNA gene sequence analysis [hereafter referred to as %Ent] and the two PCR assays [assay A and assay B]). These data were used to explore different variable constructions such as log transformation, dichotomization, and categorization. This was done to ensure that we were not missing important associations due to nonnormal distributions in the assay results or nonlinear associations with threshold effects. For the PCR assays, we examined the cycle threshold (*C_T_*) values for both the numerator and denominator, as this reflected absolute abundance.

**(ii) Unadjusted analyses.** Following initial analyses, we conducted unadjusted analysis of cases versus controls and the relative abundance of *Enterobacterales* from 16S data and the two *Enterobacterales* PCR assays via conditional logistic regression. This was repeated for the subgroups of bloodstream, respiratory, urinary tract infections, and extended-spectrum β-lactamase-producing (ESBL+) infections to assess whether the associations differed by type of infection. For the PCR assays, we also assessed the *C_T_* values, and for the numerators, we looked for interactions with the *Enterobacterales* relative abundance. We then conducted unadjusted analysis of the clinical variables versus cases and arrived at a set of clinical variables that were significantly associated with infection.

**(iii) Multivariable modeling.** These unadjusted analyses were then used to construct adjusted models that included the assays of interest (along with any significant interactions) as well as the potential clinical confounders that were significant by unadjusted analysis. This was again done by conditional logistic regression. Models were built using stepwise addition. Starting with a null model, the likelihood ratio test with a cutoff of *P <* 0.05 was used to add variables with the lowest *P* value iteratively until no additional candidates existed. If the assay of interest was not selected during this procedure, then it was forced back into the model.

The equation to model the log odds of infection conditional upon stratum *j* (a matched set of cases and controls), with independent and interacting variables was specified as follows:

$$\text{logit}\left(\text{infection}|{\text{stratum}}_{j}\right)=\overset{n}{\underset{i=1}{\sum }}\beta_{0}+\beta_{i}X_{\mathrm{ij}}+\beta_{p}X_{\mathrm{pj}}+\beta_{q}X_{\mathrm{qj}}+\beta_{\mathrm{pq}}X_{\mathrm{pj}}X_{\mathrm{qj}}$$

where *X_i_* is each independent variable (i.e., predictor) from 1 to *n* variables, *X_p_* and *X_q_* are the interacting variables, and the modeled log of the odds of infection (i.e., logit) is conditioned upon stratum *j*. If no interaction is present, the *X_p_* and *X_q_* variables are omitted. For statistical inference regarding the interaction (i.e., the *P* value), an analysis of variance (ANOVA) was performed comparing the models with/without the interaction term, *X_p_* × *X_q_*.

**(iv) Missing data.** There were many missing clinical data in our set, especially with infrequently ordered clinical laboratory results such as albumin. There were also rectal swab samples where insufficient DNA was extracted for 16S rRNA gene sequencing and PCR. Thus, prior to multivariable modeling, we applied random forest multiple imputation implemented in the R package *missForest* to fill in missing data and utilize the whole data set, as this method has shown superiority to others for building biomarker-based predictive models (20, 21).

### Data availability.

All sequence files have been deposited in the Sequence Read Archive under BioProject accession numbers PRJNA631262 and PRJNA556249.
