# Supplementary figures and images for: Enterobacterales Infection after Intestinal Dominance in Hospitalized Patients
Source: mSphere. 2020 Jul 22;5(4):e00450-20. doi: 10.1128/mSphere.00450-20 (PMC7376504; doi:10.1128/mSphere.00450-20)

A

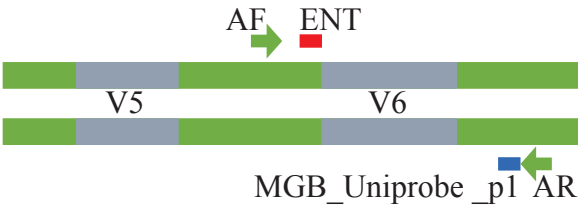

B

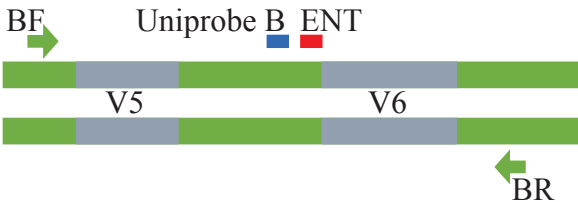

Supplement: FIG S1 [file mSphere.00450-20-sf001.pdf]

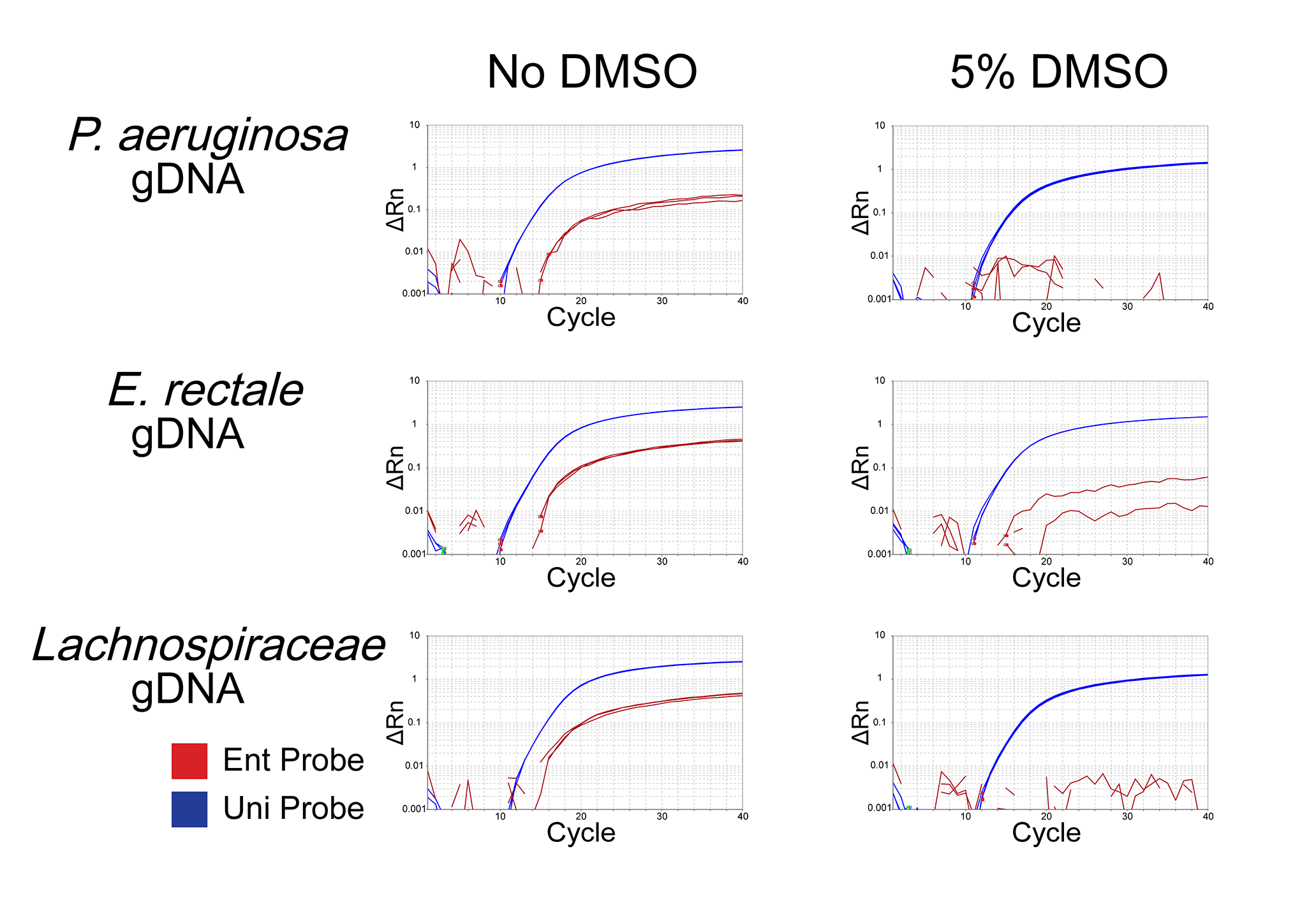

Supplement: FIG S2 [file mSphere.00450-20-sf002.tif]

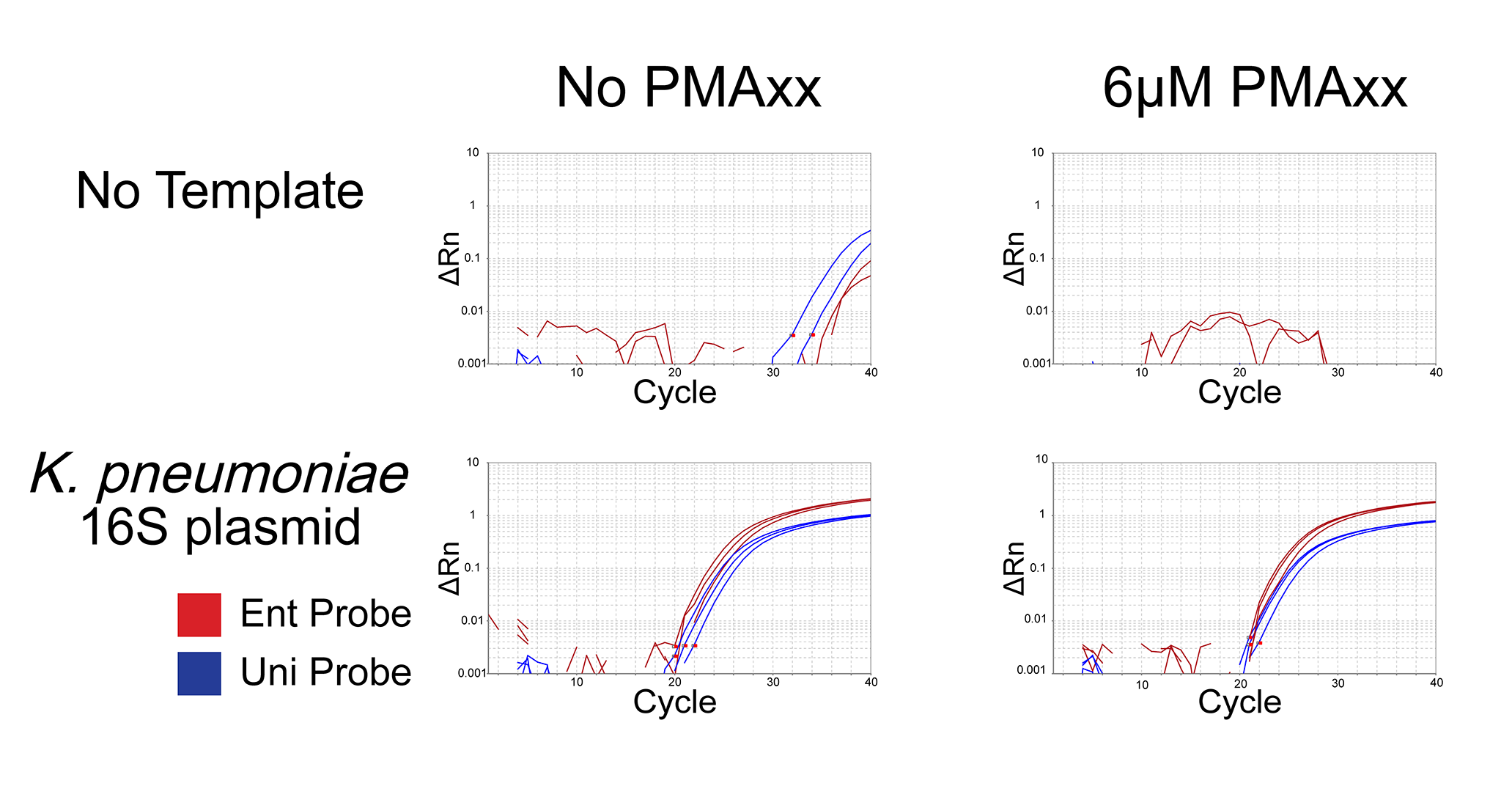

Supplement: FIG S3 [file mSphere.00450-20-sf003.tif]

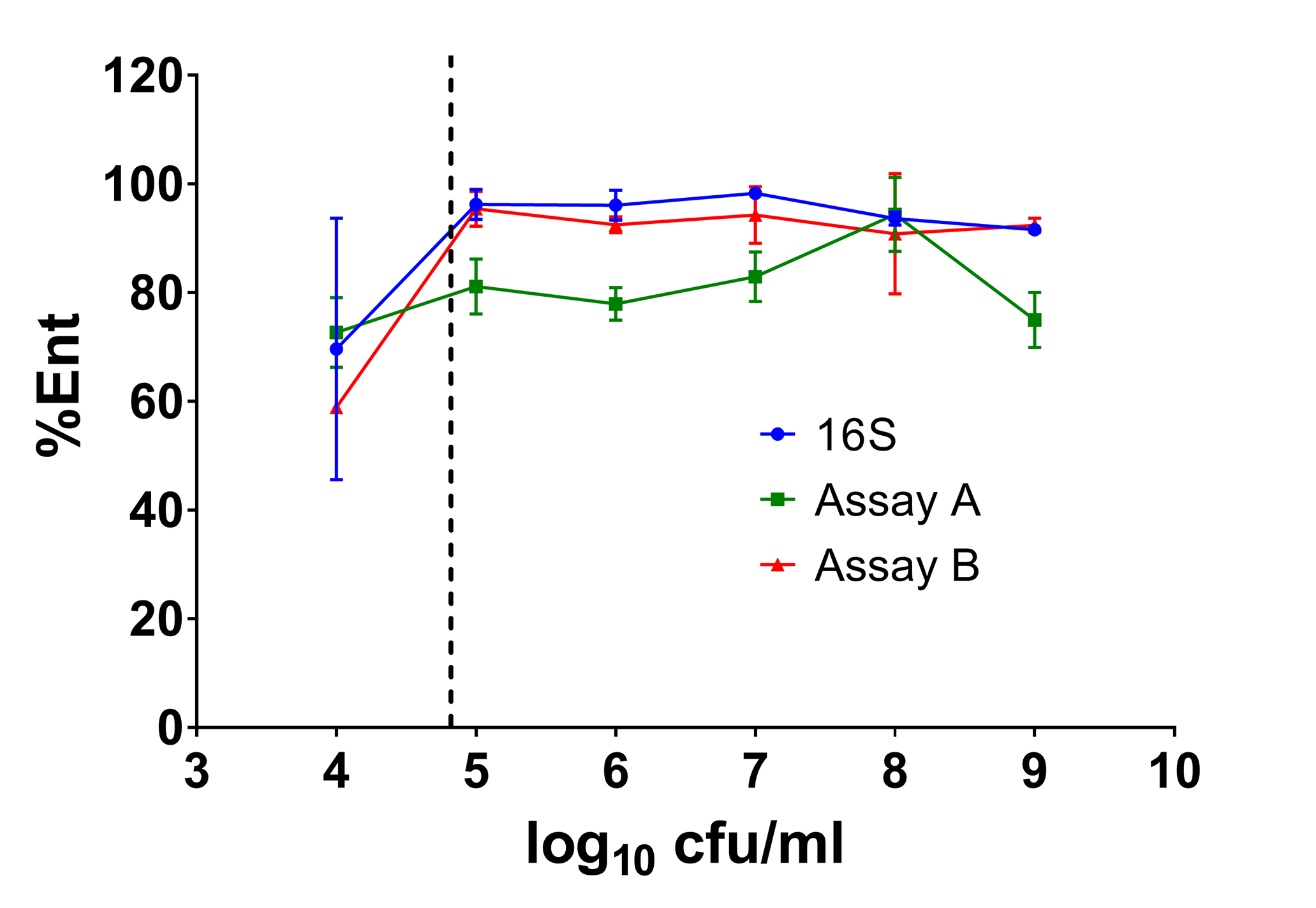

Supplement: FIG S4 [file mSphere.00450-20-sf004.tif]
