# Supplementary material for: Enterobacterales Infection after Intestinal Dominance in Hospitalized Patients
Source: mSphere. 2020 Jul 22;5(4):e00450-20. doi: 10.1128/mSphere.00450-20 (PMC7376504; doi:10.1128/mSphere.00450-20)
Supplement: TEXT S1 [file mSphere.00450-20-s0001.docx]

**Supplemental Methods**

**Media preparation for non-Enterobacteriales strains.** *Roseburia intestinalis, Eubacterium rectale*, and *Bacteroides ovatus* were grown on a modified Brain Heart Infusion media, described below:

| Chemical | Amount in 1L (g) |
| --- | --- |
| BHI | 37 |
| Yeast Extract | 5 |
| Glucose | 8 |
| L-cysteine | 0.5 |
| Vitamin K1 | 0.002 |
| Tween 80 | 0.005 |
| Haemin | 0.005 |
| 20X Salt solution* | 2.5 (ml) |

*100mL 20X salt solution: 0.5g sodium acetate, 2g sodium citrate, 0.2g Magnesium Sulfate Heptahydrate, 0.5g Manganese Sulfate Heptahydrate.

The strain *Lachnospiraceae* DW28 was grown on an enriched BHI media with 0.01% L-cysteine + 5% Fetal Bovine Serum FBS).

**Generation of DNA Library for 16S rRNA gene-based sequencing.** The V4 16S rRNA gene region was amplified from the DNA of each sample well using the dual-index sequencing methodology described in detail in the supplementary methods by Kozich et al (1). Samples were first amplified by PCR using the following reaction recipe and PCR conditions:

**Standard PCR conditions**

| Temp | Time | Cycles |
| --- | --- | --- |
| 95 | 2 m | 1 x |
| 95 | 20 s | 30 x |
| 55 | 15 s |  |
| 72 | 5 m |  |
| 72 | 10 m | 1 x |
| 4 | forever |  |

**PCR Master Mix**

| **Master Mix** | **1X reaction** |
| --- | --- |
| 10x AccuPrime PCR Buffer II | 2 uL |
| Water | 11.85 uL |
| AccuPrime HiFi Polymerase (Life Technologies, Cat# 12346094) | 0.15 uL |
| DNA | 1 uL |
| Primer set (4 uM)* prepared according to Kozich et al. | 5 uL |
| **Total volume** | **20 uL** |

In the event of low biomass samples or minimal retrieved DNA, additional DNA template was adjusted as necessary to maximize amplification effort. Alternatively, the following touchdown PCR amendment was used to amplify samples:

**Touchdown PCR Cycles**

| Temp | Time | Cycles |
| --- | --- | --- |
| 95 | 2 m | 1 x |
| 95 | 20s | 20 x  (temp increase -0.3) |
| 60 | 15s |  |
| 72 | 5m |  |
| 95 | 20 s | 20 x |
| 55 | 15 s |  |
| 72 | 5 m |  |
| 72 | 10 m | 1 x |
| 4 | forever |  |

Final PCR products were visualized using an E-Gel 96 with SYBR Safe DNA Gen Stain, 2% (Life Technologies, Cat#G7208-02). The library was normalized using the SequelPrep Normalization Plate kit (Life Technologies, Cat# A10510-01) following manufacturer’s protocol. The Kapa Biosystems Library Quantification kit for Illumina Platforms (KapaBiosystems, Cat# KK4824) was used to determine the concentration of the pooled library, and the Agilent Bioanalyzer High Sensitivity DNA Analysis kit (Cat# 5067-4626) was used to determine amplicon size. Each plate (four total) was normalized to the lowest concentrated plate, resulting in equal molar amounts of DNA from each representative plate.

**Library Preparation for Illumina MiSeq sequencing.** The MiSeq Reagent Kit V2 (500 cycles, Cat# MS-102-2003) was used to prep the library according to manufacturer’s protocol with modifications as specified in the Schloss protocol (“Preparing Libraries for Sequencing on the MiSeq, part 15039740 RevD”) for 2nM or 4nM libraries. The final load concentration of the library is 4pM, spiked with 4% PhiX to add diversity. As specified in the Schloss SOP, custom read 1, read 2, and index primers are added to the reagent cartridge. Paired end FASTQ files were generated.

**Sequencing Analysis.** The resulting sequences were processed and analyzed generally using the Schloss lab SOP using mothur v1.39.5 s (2). Explanations for each step can be found at: <http://www.mothur.org/wiki/MiSeq_SOP>. Sequences were aligned to the SILVA database (v128) (3), chimeric sequences removed using Uchime (4), and sequences were classified to the RDP database release 16 (5). Samples with less than 1000 total sequences were removed from analysis. The following specific steps were run in mothur to generate high quality sequences, classified to the order, family or genus-level:

make.contigs(file=CDCB4all.files, processors=2)

summary.seqs(fasta=CDCB4all.trim.contigs.fasta, processors=2)

screen.seqs(fasta=CDCB4all.trim.contigs.fasta, group=CDCB4all.contigs.groups, maxambig=0, maxlength=275, processors=2)

unique.seqs(fasta=CDCB4all.trim.contigs.good.fasta)

count.seqs(name=CDCB4all.trim.contigs.good.names, group=CDCB4all.contigs.good.groups)

summary.seqs(count=CDCB4all.trim.contigs.good.count_table, processors=2)

pcr.seqs(fasta=silva.seed_v128.align, start=11894, end=25319, keepdots=F, processors=2)

system(mv silva.seed_v128.pcr.align silva.v4.fasta)

summary.seqs(fasta=silva.v4.fasta, processors=2)

align.seqs(fasta=CDCB4all.trim.contigs.good.unique.fasta, reference=silva.v4.fasta, processors=2)

summary.seqs(fasta=CDCB4all.trim.contigs.good.unique.align, count=CDCB4all.trim.contigs.good.count_table, processors=2)

screen.seqs(fasta=CDCB4all.trim.contigs.good.unique.align, count=CDCB4all.trim.contigs.good.count_table, summary=CDCB4all.trim.contigs.good.unique.summary, start=1968, end=11550, maxhomop=8, processors=2)

summary.seqs(fasta=current, count=current, processors=2)

filter.seqs(fasta=CDCB4all.trim.contigs.good.unique.good.align, vertical=T, trump=., processors=2)

unique.seqs(fasta=CDCB4all.trim.contigs.good.unique.good.filter.fasta, count=CDCB4all.trim.contigs.good.good.count_table)

pre.cluster(fasta=CDCB4all.trim.contigs.good.unique.good.filter.unique.fasta, count=CDCB4all.trim.contigs.good.unique.good.filter.count_table, diffs=2, processors=2)

chimera.uchime(fasta=CDCB4all.trim.contigs.good.unique.good.filter.unique.precluster.fasta, count=CDCB4all.trim.contigs.good.unique.good.filter.unique.precluster.count_table, dereplicate=t, processors=2)

remove.seqs(fasta=CDCB4all.trim.contigs.good.unique.good.filter.unique.precluster.fasta, accnos=CDCB4all.trim.contigs.good.unique.good.filter.unique.precluster.denovo.uchime.accnos)

summary.seqs(fasta=current, count=current, processors=2)

classify.seqs(fasta=CDCB4all.trim.contigs.good.unique.good.filter.unique.precluster.pick.fasta, count=CDCB4all.trim.contigs.good.unique.good.filter.unique.precluster.denovo.uchime.pick.count_table, reference=trainset16_022016.rdp.fasta, taxonomy=trainset16_022016.rdp.tax, cutoff=80)

remove.lineage(fasta=CDCB4all.trim.contigs.good.unique.good.filter.unique.precluster.pick.fasta, count=CDCB4all.trim.contigs.good.unique.good.filter.unique.precluster.denovo.uchime.pick.count_table, taxonomy=CDCB4all.trim.contigs.good.unique.good.filter.unique.precluster.pick.rdp.wang.taxonomy, taxon=Chloroplast-Mitochondria-unknown-Archaea-Eukaryota)

The relative abundance of *Enterobacterales* was determined by calculating the percent of *Enterobacterales* (at the taxonomic order level) from all sequences.

Supplemental References

1. Kozich JJ, Westcott SL, Baxter NT, Highlander SK, Schloss PD. 2013. Development of a dual-index sequencing strategy and curation pipeline for analyzing amplicon sequence data on the MiSeq Illumina sequencing platform. Appl Environ Microbiol 79:5112-20.

2. Schloss PD, Westcott SL, Ryabin T, Hall JR, Hartmann M, Hollister EB, Lesniewski RA, Oakley BB, Parks DH, Robinson CJ, Sahl JW, Stres B, Thallinger GG, Van Horn DJ, Weber CF. 2009. Introducing mothur: open-source, platform-independent, community-supported software for describing and comparing microbial communities. Appl Environ Microbiol 75:7537-41.

3. Quast C, Pruesse E, Yilmaz P, Gerken J, Schweer T, Yarza P, Peplies J, Glockner FO. 2013. The SILVA ribosomal RNA gene database project: improved data processing and web-based tools. Nucleic Acids Res 41:D590-6.

4. Edgar RC, Haas BJ, Clemente JC, Quince C, Knight R. 2011. UCHIME improves sensitivity and speed of chimera detection. Bioinformatics 27:2194-200.

5. Wang Q, Garrity GM, Tiedje JM, Cole JR. 2007. Naive Bayesian classifier for rapid assignment of rRNA sequences into the new bacterial taxonomy. Appl Environ Microbiol 73:5261-7.
